# Livestock phenomics and genetic evaluation approaches in Africa: current state and future perspectives

**DOI:** 10.3389/fgene.2023.1115973

**Published:** 2023-06-08

**Authors:** Isidore Houaga, Raphael Mrode, Oluyinka Opoola, Mizeck G. G. Chagunda, Okeyo A. Mwai, John E. O. Rege, Victor E. Olori, Oyekanmi Nash, Cuthbert B. Banga, Tobias O. Okeno, Appolinaire Djikeng

**Affiliations:** ^1^ Centre for Tropical Livestock Genetics and Health (CTLGH), Roslin Institute, University of Edinburgh, Easter Bush Campus, Roslin, United Kingdom; ^2^ The Roslin Institute, University of Edinburgh, Easter Bush Campus, Roslin, United Kingdom; ^3^ Scotland Rural College (SRUC), Edinburgh, United Kingdom; ^4^ International Livestock Research Institute (ILRI), Nairobi, Kenya; ^5^ Department of Animal Breeding and Husbandry in the Tropics and Subtropics, University of Hohenheim, Stuttgart, Germany; ^6^ Emerge Centre for Innovations-Africa (ECI-Africa), Nairobi, Kenya; ^7^ Aviagen Limited, Newbridge, United Kingdom; ^8^ Centre for Genomics Research and Innovation, National Biotechnology Development Agency, Abuja, Nigeria; ^9^ Agricultural Research Council (ARC), Pretoria, South Africa; ^10^ Department of Animal Sciences, Faculty of Animal and Veterinary Sciences, Botswana University of Agriculture and Natural Resources (BUAN), Gaborone, Botswana; ^11^ Department of Animal Sciences, Egerton University, Egerton, Kenya; ^12^ Department of Agriculture and Animal Health, College of Agriculture and Environmental Sciences, University of South Africa, Pretoria, South Africa

**Keywords:** animal identification, livestock data recording, genetic evaluation, ICT and mobile technologies, Africa

## Abstract

The African livestock sector plays a key role in improving the livelihoods of people through the supply of food, improved nutrition and consequently health. However, its impact on the economy of the people and contribution to national GDP is highly variable and generally below its potential. This study was conducted to assess the current state of livestock phenomics and genetic evaluation methods being used across the continent, the main challenges, and to demonstrate the effects of various genetic models on the accuracy and rate of genetic gain that could be achieved. An online survey of livestock experts, academics, scientists, national focal points for animal genetic resources, policymakers, extension agents and animal breeding industry was conducted in 38 African countries. The results revealed 1) limited national livestock identification and data recording systems, 2) limited data on livestock production and health traits and genomic information, 3) mass selection was the common method used for genetic improvement with very limited application of genetic and genomic-based selection and evaluation, 4) limited human capacity, infrastructure, and funding for livestock genetic improvement programmes, as well as enabling animal breeding policies. A joint genetic evaluation of Holstein-Friesian using pooled data from Kenya and South Africa was piloted. The pilot analysis yielded higher accuracy of prediction of breeding values, pointing to possibility of higher genetic gains that could be achieved and demonstrating the potential power of multi-country evaluations: Kenya benefited on the 305-days milk yield and the age at first calving and South Africa on the age at first calving and the first calving interval. The findings from this study will help in developing harmonized protocols for animal identification, livestock data recording, and genetic evaluations (both national and across-countries) as well as in designing subsequent capacity building and training programmes for animal breeders and livestock farmers in Africa. National governments need to put in place enabling policies, the necessary infrastructure and funding for national and across country collaborations for a joint genetic evaluation which will revolutionize the livestock genetic improvement in Africa.

## 1 Introduction

African livestock sector plays an important role by contributing to the livelihoods of households as well as food, nutrition security and health. According to FAOSTAT (FAOSTAT, 2019), Africa’s total livestock population was estimated in 2018 at 2 billion poultry birds (chickens, guinea fowl, turkeys, ducks, and pigeons), 438 million goats, 384 million sheep, around 356 million cattle, 40.5 million pigs, nearly 31 million camels, and 38 million equines (donkeys, horses and mules). This livestock population comprises diverse breeds, well adapted to their environments with more than 70% under traditional production system (Ibeagha-Awemu et al., 2019), and mostly kept by the rural poor farmers. Most African livestock breeds have not been systematically improved and are characterized by low productivity. At this rate, the African livestock systems will not meet the increasing demand for animal proteins by a rapidly growing human population, urbanization and income growth (Thornton, 2010; OECD, 2018). However, in high income countries in the global North, genetic improvement has, over the past 70 years, led to dramatic gains in dairy, poultry and other commodities. To achieve these extraordinary results, structured and well-established livestock breeding programmes have been underpinned by adequate infrastructure, trained personnel, progressive farmers with access to inputs and markets. Unfortunately, the design and application of successful breeding programmes in Africa have been limited (Ibeagha-Awemu et al., 2019; Ouédraogo et al., 2021; Opoola et al., 2019; Missanjo, 2010; FAO, 2015).

Phenotypes play an important role in understanding the genetic basis of livestock performance and are essential in informing and ensuring effective herd and flock management (Mrode et al., 2020). Phenomics can be defined as the application of technologies to collect phenotypes easily, cheaply, and in large volume. Phenotypes combined with pedigree or genomic information help breeders to identify and select genetically superior animals to be parents of the next-generation, thus driving sustainable genetic improvement through genetic evaluation. Geneticists use statistical models to separate genetic effects from environmental effects by modelling the genetic effect as random with pedigree or genomic relationships between animals and modelling a herd or herd-season effects as fixed or random contemporary groups (Mrode, 2014).

Studies have shown that accuracy of predicted genetic merits of animals, and hence the genetic gains that could be realized, depend to some extent on different genetic models used for estimation (Tesfa et al., 2004; Tesfa and Garikipati, 2014; Shamia and El-Tajori, 2019; Opoola et al., 2020; Tshilate et al., 2021). Phenomics and genetic evaluation methods, and results obtained, are therefore fundamental at farm level for profitability; at the national level for effective agricultural policies formulation, and at the continental level for across country collaboration for livestock improvement (Mrode et al., 2020).

However, in most African countries, phenotyping or performance recording has always been a major challenge due limited investments in on-farm recording (Trivedi, 1998; Ojango et al., 2017; Tshilate et al., 2021). Livestock performance recording and genetic evaluation have been initiated and are on-going in a limited number of countries in Africa, with different levels of success and rigour. However, the current status of livestock performance recording, the availability of data, systems of management of such data, the genetic evaluations methods used as well as the challenges faced by such efforts are yet to be fully documented. Better understanding of the key factors that affect phenomics and genetic/genomic evaluations in African countries will inform the development of mitigation strategies as well as the design of livestock breeding programmes that meet the current and future needs of the continent.

The aim of this study was therefore to assess the current status of phenomics and other data systems and recording, livestock genetic evaluation approaches in Africa and to demonstrate the effects of various genetic models on the accuracy and rate of genetic gain that could be achieved in livestock.

## 2 Materials and methods

### 2.1 Data collection

#### 2.1.1 Survey

A survey was carried out in 2020 using an online questionnaire, telephone and skype interviews. The questionnaire recipients included: 1) current Food and Agriculture Organization of the United Nations (FAO) national focal points for animal genetic resources, 2) individuals identified from lists of participants of scientific conferences and workshops on livestock production and genetics held in Africa from 2000 to 2019. The conferences and workshops were randomly selected and all the names on the delegate lists were contacted as respondents. The survey questionnaire was sent to 501 respondents from the 54 African countries. The contacted delegates were predominantly scientists and other professionals in animal/veterinary science, animal breeders and geneticists affiliated with government livestock ministries, research institutes, universities, farmers associations and NGO’s . The e-survey was active for a period of 68 days. A reminder e-mail was sent every 15 days after first dispatch. The survey questions were a combination of open-ended, close-ended, structured and unstructured questions (Supplementary Data Sheet S1). The main themes in the survey included: livestock species, genotypes (breeds and crossbreeds) in use, human capacity in animal science, livestock genetic improvement initiatives, data recording and animal ranking systems, genetic evaluation methods being used and challenges affecting livestock genetic evaluations, the available livestock data and if the custodians of such data are willing to be supported to use the same data to undertake better genetic evaluation and multi-country collaborations for livestock genetic improvement in Africa. Responses were obtained from 92 respondents in 38 African countries across the five regions. Northern Africa (Algeria, Egypt, Mauritania, Morocco, and Tunisia), Central Africa (Cameroon, Chad, Congo, Democratic Republic of Congo, Gabon), Southern Africa (Angola, Malawi, Mozambique, South Africa, Zambia, and Zimbabwe), Eastern Africa (Comoros, Djibouti, Ethiopia, Kenya, Rwanda, Seychelles, Sudan, Tanzania, and Uganda) and Western Africa (Benin, Burkina Faso, Cabo Verde, Côte d'Ivoire, The Gambia, Ghana, Liberia, Mali, Niger, Nigeria, Senegal, Sierra Leone, and Togo) (Supplementary Table S1).

#### 2.1.2 Joint genetic analysis

The data used for joint genetic evaluation was reported by the respondents. Thus, performance and pedigree data were obtained from the Agricultural Research Council (ARC) in Pretoria, South Africa and the Kenya Livestock Breeders Association (KLBA), Kenya. In summary, the data comprised 305-day milk yield (MY305) records from 2,333 and 25,208 first lactation of Holstein-Friesian cows in Kenya (1979–2014) and South Africa (1997–2014), respectively. The pedigree data comprised 103 and 505 sires with daughter performance records in Kenya and South Africa, respectively. The common sires between both countries were 40. The reproduction traits were age at first calving (AFC, months) and the interval between first and second calving (CI1, months).

### 2.2 Statistical analysis

Survey data were analysed by country and region using descriptive statistics tools of the R software package (R Core Team, 2013). Proportions were estimated for each of the studied variables in the five regions specified. The proportions were averaged over regions without considering any heterogeneity among respondents within a given region. The Chi-square test was used to test for differences between observed proportions in the five African regions. Z-test was used for pairwise comparison between proportions.

In the joint-genetic analysis, a multi-trait animal model was used in Blupf90 (Misztal et al., 2018) to analyse first lactation MY305, AFC and CI1 within country using the model*:*

$$Y_{ijk}=H_{j}+HYS_{k}+age\;\left(cov\right)+a_{i}+e_{ijk}$$ (1)

Where: *Y*_*ijk*_ = an observation of MY305, AFC or CI1; *H*_*j*_ = fixed effect of herd j in which animal i was born; *HYS*_*k*_ = fixed effect of the *k*th herd-year-season of production; *age (cov)* = age as covariate; *a*_*i*_ = random additive genetic effect of animal i; and *e*_*ijk*_ = random error term. The calving age was not included in the analysis of AFC. The across-country analysis was implemented by pooling data from the two countries using the model similar to Eq. 1 with an additional fixed effect of country. Genetic gains per generation were predicted for each country and trait by using the Breeders’ equation (Falconer and Mackay, 1996): R = i. ρ. σg.

Where R: Response to selection or predicted genetic gain per generation, i: Selection intensity, ρ: Accuracy of selection (square root of reliability of sire EBVs), σ g: genetic standard deviation of studied traits. The R was based on sire selection only within- and across country (Rendel and Robertson, 1950). Different selection intensities were tested based on selection of the top 5, 10, 25, 50, 75, and 100 sires within- and across-country. Predicted levels of genetic gain achievable within-country was compared to the predicted genetic gains across-countries.

## 3 Results

### 3.1 Current state of animal identification and data recording in Africa

At the herd level, Ear tagging (33.1%) was the most used animal identification method (*p* < 0.05) followed by branding (17.6%), ear notching (16.3%), ear tattooing and number tagging (11%) while the tribal signs (0.4%) and hindquarter tattooing (0.4%) were the least frequent animal identification methods. Only 18.5% of the respondents mentioned existence of national animal identification system (NAIS) in their respective countries (Egypt, Morocco, Mozambique, Nigeria, South Africa, Tanzania, Tunisia and Zimbabwe). No NAIS was mentioned in Central Africa. However, most of the mentioned NAISs are still rudimentary based on ear tags. Furthermore, it was reported that farmers are not interested to participate in the national animal identification system. The NAISs reported by the respondents are described below.

In Egypt, animal identification is limited to state organisations and farms. This is part of the preservation of the purebred and developed chickens in some governmental institutional stations and projects (*in vivo*) and in National Gene Bank (*in vitro*). In Morocco, the electronic chips identification for goat and sheep is managed by the ministry of agriculture. All goat and sheep farmers are recommended to use E-chips for identification of their animals. Horses are ear-tagged. In Tunisia, two NAISs were reported: the identification system for cattle, sheep, goat and camel based on ear tagging and managed by “Office de l' Elevage et des Pâturages (OEP)” and the identification system for horses based on electronic chips and managed by The National Foundation for the Improvement of The Horses Breed (FNARC). In Nigeria, the animal identification system is not well developed. However, there is a recent Livestock24 programme (https://livestock247.com/) that tracks animals from farm, livestock market and slaughter houses in Nigeria. In Tanzania**,** the Tanzania National Animal Identification and Traceability System (TANLITS; https://asdp.kilimo.go.tz/) has been recently developed. Thus, ear tagging or branding of unique national ID has been introduced but is yet to be practiced by all farmers. TANLITS is a web-based platform developed to drive the animal identification, registration and traceability Act Chapter 184 of the Tanzania laws and its regulations. The purposes of TANLITS include controlling livestock theft and animal diseases, to regulate movement of livestock, enhancing food safety assurance and promote access to livestock markets. In South Africa, systematic animal identification of cattle was reported by respondents. The Department of Agriculture, Land and Rural Development manages the South African NAIS. Farmers are given a branding criterion and from age of 7 months any cattle born in South Africa needs to be branded. The South Africa Studbook Association also manage animal identification through the registration and recording of the birth and ownership information of purebred animals and continuously update these animals’ pedigree information. In Mozambique, a national ear tag identification system was reported where a combination of letters (indicating year) and numbers indicating order of birth in the group are used to tag animals. Some units also use tattoos. However, the majority of farmers in Mozambique do not identify their animals. In Zimbabwe, a NAIS exists for Tuli cattle and is managed by the Livestock Identification Trust (LIT). Farms pay for tag and the LIT generates tag numbers. Tags give traceability and are specific to each farm. Each region with its own brand and each farm within a region with its own ID. The reported livestock data in Africa and the custodians are described below.

**FIGURE 1:**
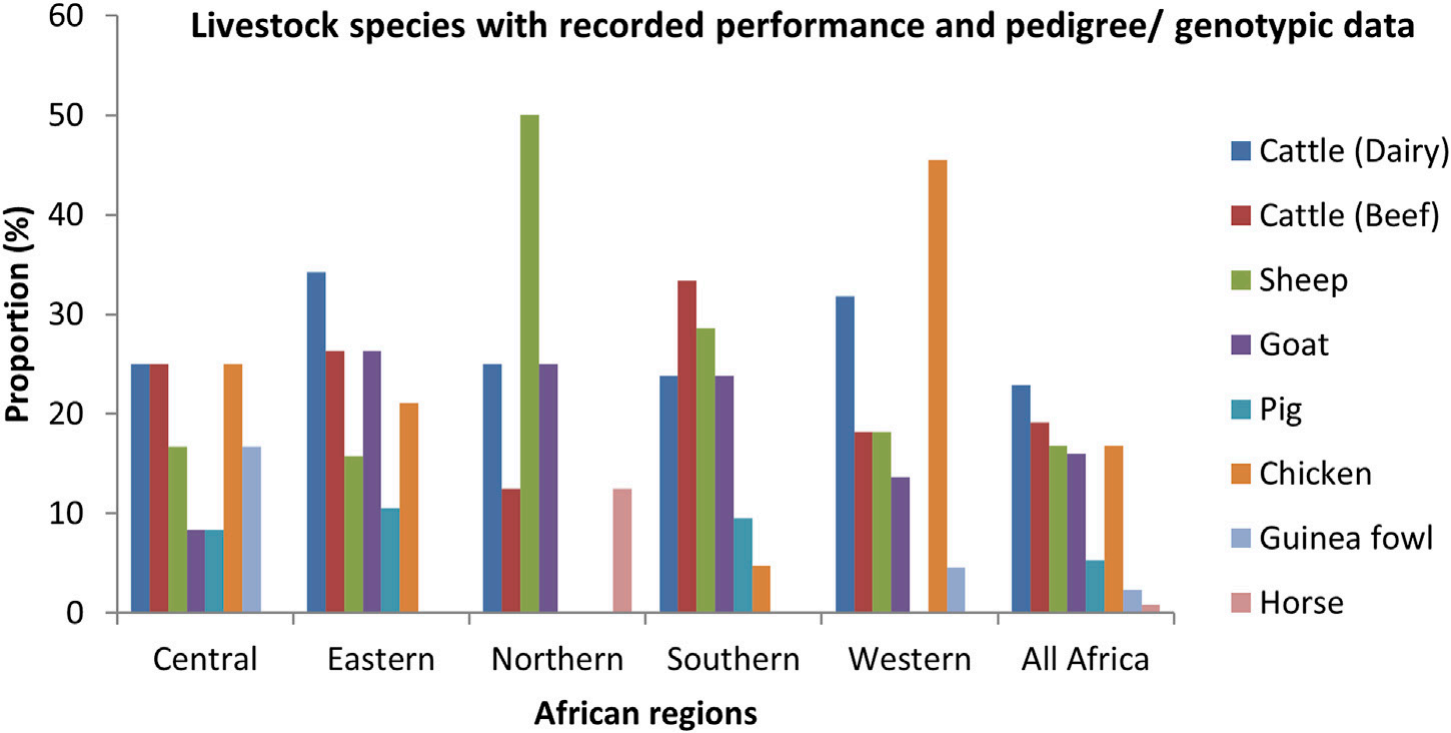
Proportion of livestock species with both performance and pedigree/genotypic data recorded (*p* < 0.01).

From the survey, 58.7% of respondents mentioned ongoing livestock genetic improvement programmes or projects with performance and pedigree and/or genotypic data available in their respective countries (Algeria, Benin, Burkina Faso, Cameroon, Chad, Côte d'Ivoire, Djibouti, DR Congo, Egypt, Ethiopia, Kenya, Malawi, Mali, Morocco, Niger, Nigeria, Rwanda, Senegal, South Africa, Sudan, Tanzania, The Gambia, Togo, Zambia, and Zimbabwe). Out of the positive responses, Eastern Africa (76.2%) and Southern Africa (75%) had the highest proportions of ongoing genetic improvement initiatives followed by Central Africa (60%), Western Africa (52.6%) and the Northern Africa (45.4%) had the lowest (*p* < 0.01). The available data per species and regions are shown in Figure 1.

**FIGURE 2:**
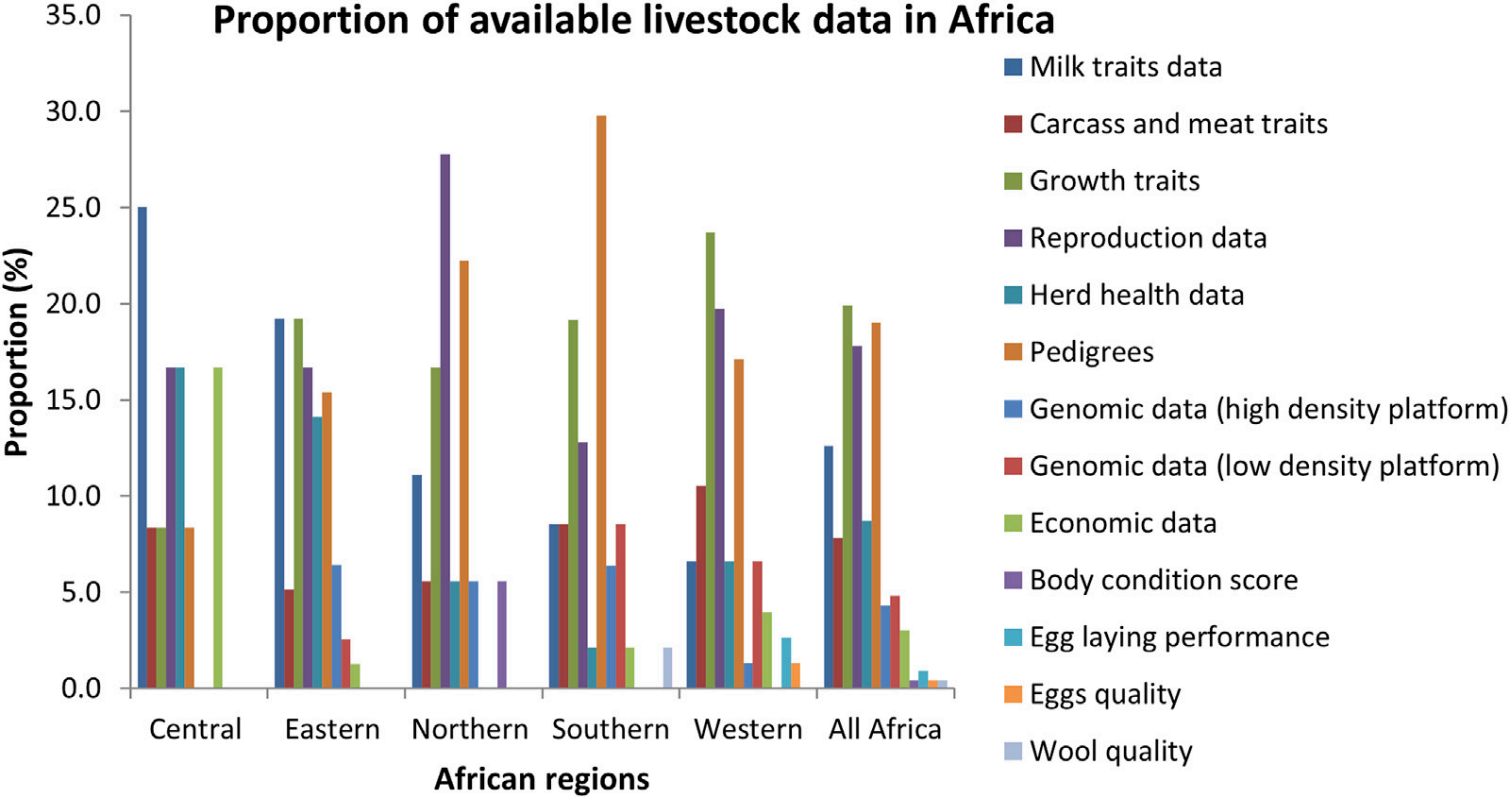
Proportion of available livestock data in Africa (*p* < 0.001).

The reported data were recorded mostly on dairy cattle (22.9%) followed by beef cattle (19.1%), chicken (16.8%), sheep (16.8%), goats (16%), pigs (5.3%), guinea fowl (2.3%) and horses (0.8%). In Western Africa, chicken was the most reported specie with production and pedigree data while dairy cattle, beef cattle and chicken were the most reported in Central Africa. Beef cattle was the most reported in Southern Africa. Dairy cattle and sheep were the most reported in Eastern and Northern Africa respectively. As shown in Figure 2, the most recorded data in Africa were on growth traits (19.9%), followed by pedigrees (19%), reproduction data (17.8%), milk traits data (12.6%), herd health data (8.7%), carcass and meat traits data (7.8%), low density genomic data (4.8%), high density genomic data (4.3%), economic data (3%) and egg laying performance (0.9%). Body condition score, eggs quality and wool quality were the least recorded traits (0.4%). There were significant differences across traits (*p* < 0.001). The most recorded traits in Western Africa were growth traits (23.7%) followed by reproduction traits (19.7%) and pedigree data (17.1%). In Eastern Africa, milk traits (19.2%), growth traits (19.2%), reproduction data (16.7%) and pedigrees (15.4%) were the most reported. In Northern Africa, reproduction data (27.8%), pedigree (22.2%) and Growth traits (16.7%) were the most reported while pedigree data (29.8%), growth traits (19.1%) and reproduction data (12.8%) were the most reported in Southern Africa. In Central Africa, milk traits (25%), reproduction data (16.7%), health data (16.7%) and economic data (16.7%) were the most reported.

**FIGURE 3:**
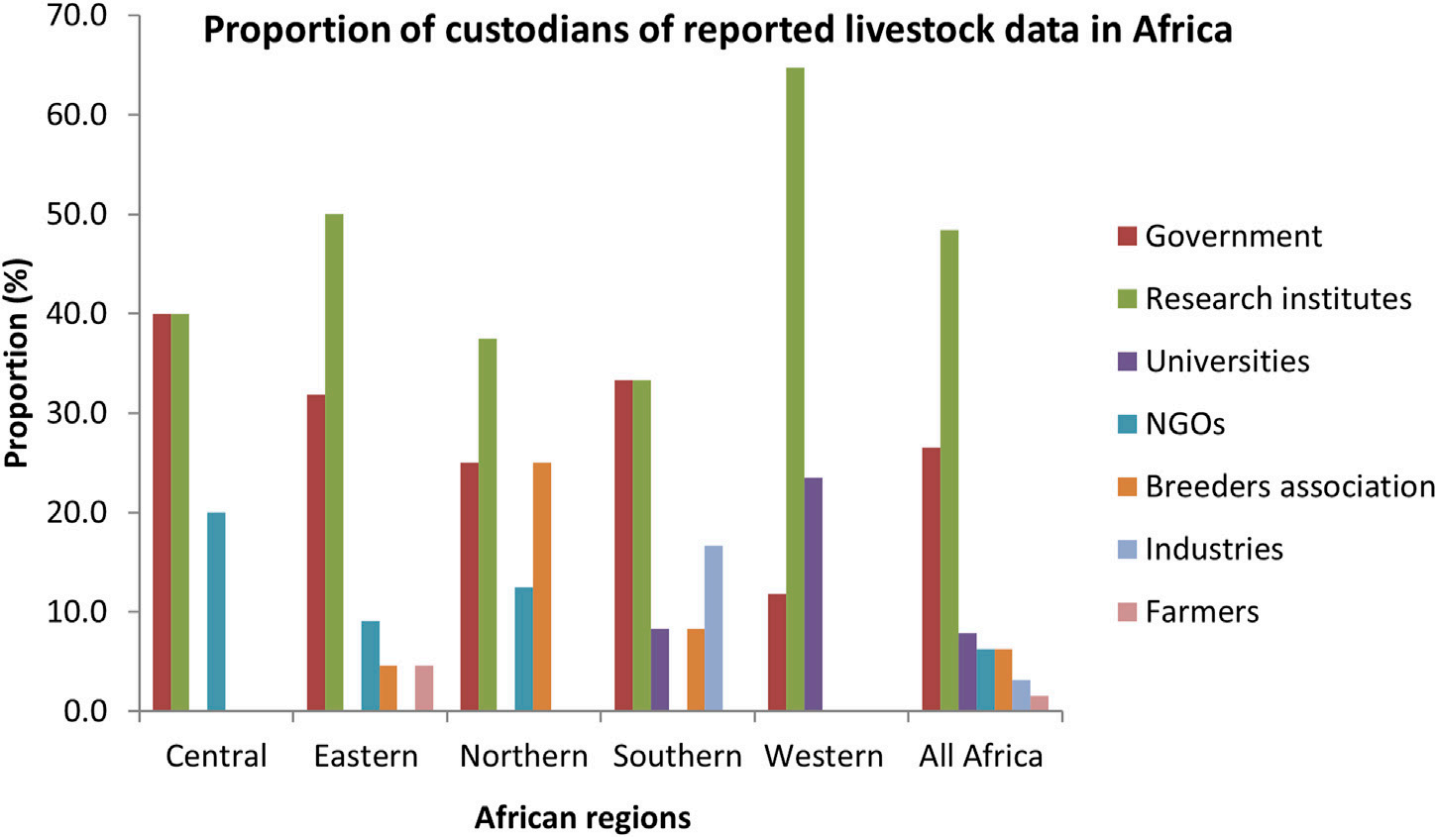
Custodians of reported livestock data in Africa (*p* < 0.001).

The most important custodians of the reported data in Africa were research institutes (48.4%) followed by government (26.6%), universities (7.8%), NGOs (6.3%), breeders/farmers association (6.3%), industries (3.1%) and individual farmers (1.6%), with significant differences across custodians (*p* < 0.001) as shown in Figure 3. In Western, Eastern and Northern Africa, research institutes were the most important custodians. In Central and Southern Africa, the available data were equally detained by research institute and government.

### 3.2 Genetic evaluation and animal ranking approaches in Africa

**FIGURE 4:**
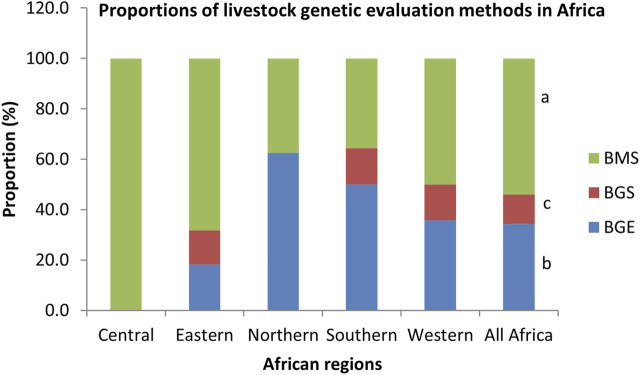
Proportions of livestock genetic evaluation methods in Africa. BGE, Based on Genetic evaluation; BGS, Based on Genomic evaluation; BMS, Based on mass selection; ^a,b,c^ *p* < 0.001.

The livestock genetic evaluation methods being used in Africa are shown in Figure 4. The selection methods being used are mostly based on mass selection based on phenotypic data (53.9%) followed by genetic evaluation, including pedigree information (34.3%) and genomic evaluation (11.8%). There were significant differences between methods (*p* < 0.001). A similar trend was observed within each region, except in Central Africa where selection was based only on phenotypic performance data. Most respondents (90.7%) who have reported availability of livestock data believed that it is a good idea to share such data. Out of this number, 74.1% opined that the custodians of the reported data would be willing to be supported to use the same data to undertake better genetic evaluations. However, some (25.9%) of the respondents believe that the custodians may not be willing to be supported.

Only 13% (12 out 92) of the respondents mentioned ongoing national animal ranking systems (NARS) in their respective countries (Côte d'Ivoire, Morocco, Niger, Nigeria, South Africa, Tanzania, Kenya, Tunisia, Uganda, and Zimbabwe) as presented in Table 1. No NARS was reported by Central African countries. The reported NARSs were based on genetic evaluation (77.8%), mass selection based on phenotypic performance data (66.7%) and genomic evaluation (22.2%). In Côte d'Ivoire and Niger, the national animal ranking systems are based solely on performance data (growth and milk traits). In Morocco, the best horses are ranked at the national level by either considering phenotypic performance or by integrating the pedigree (genetic) information. South Africa has a more advanced NARS where cattle are ranked using quantitative genetic models that integrate pedigree information and the genomic data to some extents. In Tanzania, the national animal ranking system for cattle is based on performance, pedigree, and genomic evaluation especially for dairy cattle. In Kenya, the Kenya Animal Genetic Resources Centre (KAGRC) does regular bull evaluations using performance and pedigree information. The national animal ranking system in Tunisia focused on cattle by using pedigree-based best linear unbiased prediction (BLUP) models. The animals in the Herdbook database in Zimbabwe are ranked using pedigree-based breeding value (EBVs) and this is mainly for the Zimbabwean Tuli Beef breed. In Uganda, it was reported that performances and pedigree data of individual animals are recorded over a period of time. The animals are then ranked either based on the phenotypic performance data only or pedigree information combined with performance data. The national animal ranking system in Nigeria was not described by the respondents. They believe that the information can be obtained from the National Animal Production Research Institute (NAPRI) or from the Federal Ministry of Agriculture and Rural Development (FMARD) in Nigeria.

**TABLE 1:** Description of reported national animal ranking systems in Africa.

Country	African region	Species	Ranking methods	Characteristics of the ranking system
Côte d'Ivoire	Western	Cattle (Beef), Sheep and Goat	Based on mass selection	Based on the performances data, the best animals are selected
Niger	Western	Cattle (Dairy), Cattle (Beef), Sheep, Goat and Chicken	Based on mass selection	Selection based on mass selection for growth and milk traits
Nigeria	Western	Cattle (Dairy), Cattle (Beef), Sheep and Goat	Based on mass selection and based on genetic evaluation	Ranking system not described by respondents. They believed that the information is domiciled with National Animal Production Research Institute (NAPRI), FMARD, Kaduna, Nigeria
Uganda	Eastern	Cattle (Dairy), Cattle (Beef), Goat, Pig and Chicken	Based on genetic evaluation, and based on mass selection	Performances of individuals animals are recorded over time. Samples are taken to determine genetic profiles of the animals under evaluation. The animals are then ranked either based on the phenotypic performance or genetic information
Tanzania	Eastern	Cattle (Dairy)	Based on mass selection, based on genetic evaluation and based on genomic evaluation and	Ranking of animals based on the genomic, genetic and phenotype data
Zimbabwe	Southern	Cattle (Beef)	Based on genetic evaluation	The animals in the Herdbook database are ranked using pedigree-based breeding value (EBVs). The national ranking system is more based on the Tuli Beef breed
Kenya	Eastern	Cattle (Dairy), Cattle (Beef)	Based on mass selection and based on genetic evaluation	The Kenya Animal Genetic Resources Centre (KAGRC) does regular bull evaluations using performance and pedigree information
Morocco	Northern	Horse	Based on genetic evaluation, and based on mass selection	Depending on the use of the horse, the best animals are ranked by either considering phenotypic performance or by integrating the pedigree (genetic) information
Tunisia	Northern	Cattle (Dairy)	Based on genetic evaluation	Cattle ranking based on pedigree-BLUP
South Africa	Southern	Cattle (Dairy), Cattle (Beef), Sheep, Goat and Pig	Based on genetic evaluation, and Based on genomic evaluation	Animals are ranked by using quantitative and Molecular genetics. The methods used include pedigree, genomics and performance data

### 3.3 Potential for multi-country genetic evaluation and impact on genetic progress

The potential for multi-country genetic evaluation in Africa was assessed through the survey. Most of the respondents (92.4%) believe that across-country genetic evaluation will have some mileage in improving livestock production in Africa. According to these respondents, the reasons for a potential success in across-country genetic evaluation included 1) sharing of resources and benefits (42.4%), 2) existence of transboundary breeds across countries and regions (34.1%), 3) large reference population will give high accurate predictions making genomic selection possible (15.3%), 4) similar environmental conditions and breeding challenges across countries (8.2%), 5) high and fast genetic gain (4.7%), 6) existing high livestock genetic diversity in Africa (4.7%), 7) capacity of African diaspora already involved in across country genetic evaluation (3.5%) and 8) willingness to implementing multi-country genetic evaluation in Africa (1.2%) were expressed. However, some of the respondents (7.6%) mentioned that multi-country genetic evaluation would not work in the African livestock production systems, mainly because of: 1) limited genetic material exchange and hence lack of herd connectedness between countries (42.9%), 2) lack of functional national animal identification, data recording and evaluation systems (42.9%), 3) lack of required infrastructure for across country genetic evaluation (28.6%), 4) limited international collaboration (ICAR, Interbull) in Africa (14.3%), 5) lack of human capacity (14.3%), 6) lack of enabling breeding policies for across country genetic evaluation (14.3%) and 7) scepticism and concerns about data sharing (14.3%). Two respondents mentioned that multi-country genetic evaluation for poultry, pigs and small ruminants can be challenging, costly and not feasible.

Using the reported dairy performance and pedigree data from Holstein-Friesian in South Africa and Kenya, we assessed the impact of different genetic evaluation approaches and models, on accuracy and the rate of genetic progress that could be achieved for 305-day milk yield (MY305), age at first calving (AFC) and first calving interval (CI1) (Tables 2, 3). The results showed that the accuracies of prediction in multi-traits across-country genetic evaluation were higher than the accuracies of within country genetic evaluation for MY305 (0.7 vs. 0.56) and AFC (0.78 vs. 0.49) in Kenya and for AFC in South Africa (0.78 vs. 0.76) (Table 3). Regardless of proportion of selected sires (Top 5–100 sires), selection based on multi-country genetic evaluation resulted in higher and favourable gains for MY305 in first lactation in Kenya and for AFC in both Kenya and South Africa. Selection based on the top 50 to 100 sires in across-country genetic evaluation resulted in highest responses for all the studied traits in South Africa (Table 3). Kenya would only achieve 4%–73% and 3%–52% of genetic responses respectively for MY305 and AFC from within country genetic evaluations compared to multi-country. This translates to a benefit of 27%–96% and 48%–97% respectively for MY305 and AFC. Furthermore, South Africa would only achieve 63%–66% and 88%–92% of genetic responses respectively for AFC and CI1 from within country genetic evaluations compared to a multi-country. This translates to a benefit of 34%–37% and 8%–12% respectively for AFC and CI1. Kenya would benefit more from multi-country genetic evaluation of MY305 and AFC compared to South Africa.

**TABLE 2:** Predicted genetic gain (PGG) per generation from sire selection only (i.e., the top 5–25 sires are selected) for 305-day milk yield (MY305, Kg), age at first calving (AFC) and first calving interval (CI1) in first lactation from multi-trait within- and across-country genetic selection (Holstein-Friesians) in Kenya (KE) and South Africa (SA) Top 5–25 sires.

Top 5 sires															
Trait	KE			PGG	%PGG	SA					Multi-country			PGG	%PGG
	*i*	σ g	ρ			i	σ g	ρ	PGG	%PGG	*i*	σ g	ρ		
MY305	2.08	634	0.56	739.86	73.13	2.67	524.65	0.73	1,024.80	101.30	2.73	528	0.70	1,011.67	100
AFC	2.08	68	0.49	69.02	52.89	2.67	43.02	0.76	86.76	66.48	2.73	61.19	0.78	130.50	100
CI1	2.08	104	0.56	120.96	405.26	2.67	18.49	0.56	27.71	92.83	2.73	20.29	0.54	29.85	100

Top 10 sires															
Trait	KE			PGG	%PGG	SA					Multi-country			PGG	%PGG
	*i*	σ g	ρ			i	σ g	ρ	PGG	%PGG	*i*	σ g	ρ		
MY305	1.77	634	0.56	629.21	68.16	2.42	524.65	0.73	930.59	100.81	2.49	528	0.70	923.14	100
AFC	1.77	68	0.49	58.70	49.29	2.42	43.02	0.76	78.78	66.16	2.49	61.1948	0.78	119.08	100
CI1	1.77	104	0.56	102.87	377.70	2.42	18.49	0.56	25.16	92.38	2.49	20.2946	0.54	27.24	100

Top 25 sires															
Trait	KE			PGG	%PGG	SA					Multi-country			PGG	%PGG
	*i*	σ g	ρ			*i*	σ g	ρ	PGG	%PGG	*i*	σ g	ρ		
MY305	1.29	634	0.56	458.86	57.88	2.07	524.65	0.73	794.51	100.22	2.14	528	0.70	792.79	100
AFC	1.29	68	0.49	42.81	41.86	2.07	43.02	0.76	67.26	65.77	2.14	61.1948	0.78	102.26	100
CI1	1.29	104	0.56	75.02	320.73	2.07	18.49	0.56	21.48	91.84	2.14	20.2946	0.54	23.39	100

**PGG**: Predicted genetic gain per generation (Response to selection). ***i***: Selection intensity. **ρ**: Accuracy of selection (square root of reliability of EBVs). ***σ g***: Square root of trait genetic variance estimate.

**TABLE 3:** Predicted genetic gain (PGG) per generation from sire selection only (i.e., the top 50–100 sires are selected) for 305-day milk yield (MY305, Kg), age at first calving (AFC) and first calving interval (CI1) in first lactation from multi-trait within- and across-country genetic selection (Holstein-Friesians) in Kenya (KE) and South Africa (SA) Top 50–100 sire.

Top 50 sires															
Trait	KE			PGG	%PGG	SA					Multi-country			PGG	%PGG
	*i*	σ g	ρ			i	σ g	ρ	PGG	%PGG	*i*	σ g	ρ		
MY305	0.82	634	0.56	291.68	42.56	1.76	524.65	0.73	675.52	98.56	1.85	528	**0.70**	**685.36**	**100**
AFC	0.82	68	0.49	27.21	30.78	1.76	43.02	**0.76**	57.19	64.69	1.85	61.1948	**0.78**	**88.41**	**100**
CI1	0.82	104	0.56	47.69	235.83	1.76	18.49	0.56	18.27	90.33	1.85	20.2946	0.54	**20.22**	**100**

Top 75 sires															
Trait	KE			PGG	%PGG	SA					Multi-country			PGG	%PGG
	*i*	σ g	ρ			i	σ g	ρ	PGG	%PGG	*i*	σ g	ρ		
MY305	0.46	634	0.56	163.62	26.77	1.56	524.65	0.73	598.76	97.95	1.65	528	0.70	611.27	100
AFC	0.46	68	0.49	15.26	19.36	1.56	43.02	0.76	50.69	64.29	1.65	61.1948	0.78	78.85	100
CI1	0.46	104	0.56	26.75	148.33	1.56	18.49	0.56	16.19	89.77	1.65	20.2946	0.54	18.03	100

Top 100 sires															
Trait	KE			PGG	%PGG	SA					Multi-country			PGG	%PGG
	*i*	σ g	ρ			i	σ g	ρ	PGG	%PGG	*i*	σ g	ρ		
MY305	0.07	634	0.56	24.85	4.45	1.41	524.65	0.73	540.02	96.76	1.51	528	0.70	558.10	100
AFC	0.07	68	0.49	2.33	3.24	1.41	43.02	0.76	46.10	63.96	1.51	61.1948	0.78	72.08	100
CI1	0.07	104	0.56	4.08	24.64	1.41	18.49	0.56	14.60	88.23	1.51	20.2946	0.54	16.55	100

**PGG**: Predicted genetic gain per generation (Response to selection). ***i*** : Selection intensity. ***ρ*** : Accuracy of selection (square root of reliability of EBVs). **σ** ***g*** : square root of trait genetic variance estimate.

### 3.4 Challenges in livestock phenomics and genetic evaluations in Africa

Respondents identified the main challenges affecting livestock data recording and genetic evaluation in Africa. The challenges varied across countries and regions. At the continental level, the following challenges were cited: 1) lack and/or inadequate human capacity and skills in livestock genetic evaluation (60.9%),2) lack of required infrastructure for livestock data recording and genetic evaluation (42.4%), 3) inadequate and lack of governmental funding for livestock genetic evaluation (39.1%), 4) lack of enabling animal breeding policies (32.6%), 5) poor animal identification and data recording (23.9%), and 6) lack of systematic animal performance and pedigree data recording (22.8%) among others. There was significant differences in the reported challenges across the five African regions (*p* < 0.05). The lack of human capacity in livestock genetic evaluation was the main challenge in Eastern Africa (85.7%), Southern Africa (66.7%) and Western Africa (60.5%). The major challenge in Northern Africa and Central Africa were poor animal identification and data recording (54.5%) and lack of government funding for livestock data recording and genetic evaluation (60%), respectively. The respondents mentioned the following challenges to potential implementation of multi-country genetic evaluation in Africa: 1) human capacity for multi-country genetic evaluation and management (28.4%) 2) differences in national breeding policies (26.1%), 3) lack of framework for joint funding for such an initiative (21.6%),4) required infrastructure for across country genetic evaluation (20.5%), 5) absence of standardization of data collection tools and methods (17%),6) differences in breeding goals/traits of interests among countries (15.9%) and 7) data access and benefits sharing issues across countries (14.8%), among others.

## 4 Discussion

This study has highlighted, for the first time, the current state of livestock phenomics and genetic evaluation approaches in Africa, the main challenges at the national, sub-regional and continental levels. From this, possible solutions can be inferred. We further demonstrated that the multi-country genetic evaluation in South African and Kenyan Holstein-Friesian cattle increased accuracy of prediction and the expected genetic gains. The major challenges highlighted by the current study were related to animal identification and data recording systems, human capacity to analyse the data, infrastructure, funding, and animal breeding policies. Similar issues have been previously reported in dairy cattle and other livestock species in Africa (Zonabend et al., 2013; Wurzinger et al., 2014; Ibeagha-Awemu et al., 2019; Okpeku et al., 2019; Opoola et al., 2019; Rege et al., 2022). Although most of the respondents described themselves as experts in the livestock sector, it is important to acknowledge that some of them may not be fully aware of the reality in the field, and the increasingly available tools and methods.

### 4.1 Current state of phenomics and genetic evaluation approaches in Africa

Systematic animal identification and routine capture of livestock production and pedigree data are necessary for the prediction of relative genetic merits of animals and provide the required information needed to inform herd management and improvement (Mrode et al., 2020). This study has confirmed that, in many of the African countries, animal identification, pedigree and performance recording systems are generally absent (van Marle-Köster et al., 2014). In the African smallholders and even commercial production systems, data collection and storage still pose great challenges (Ibeagha-Awemu et al., 2019). Thus, it will be difficult to identify high genetic merit individuals that are both highly adapted to smallholder farmers’ systems and have optimal productivity for the environment. Some performance recording and genetic evaluation programmes exist in African smallholder farming systems. These include the African Dairy Genetic Gains Programme (ADGG) that routinely collect on-farm herd health, milk production traits and genetic information on dairy cattle in Tanzania, Ethiopia, Kenya, Uganda and Rwanda and digitally shares feedback of the findings from the collected data to farmers (ILRI, 2021). The ADGG programme has successfully implemented genomic selection in pure and crossbred dairy cattle in Eastern Africa (Marshall et al., 2019; Burrow et al., 2021; Mrode et al., 2021) and this could be extended to other African countries and regions. This will require important genomic and performance data generation across the continent. The reasons for successful genomic selection by ADGG include closer engagement of the farmers and co-definition of the problems, hence co-ownership of the strategies to solving the problems of lack of access to appropriate dairy seedstocks. Secondly, immediate use of the results from analyses and sharing these with the farmers help to inform their herd managements and profitability. The farmers therefore see the relevance of the recording and their roles in the generation of the data. First initiated in the year 2016, ADGG is relatively young, and it is still benefiting from donor support. Although much thought has been given to sustainability considerations, there is still uncertainty on what will happen when the donor support stops.

As observed in the present study, South Africa has a well-established cattle data recording and ranking system. It has been previously reported that South Africa is the only sub-Saharan Africa country having a sustainable national animal identification and performance recording scheme, as well as routine genetic evaluation programmes (Ramatsoma et al., 2015). South Africa could therefore serve as a model for other African countries for national animal identification and ranking systems. It must be emphasized that the systems in South Africa are focused on and driven by (large and medium) commercial farmers. The country could, therefore, benefit from collaborations with countries which have developed genetic evaluation models geared to and/or inclusive of smallholders. Other cases of successful animal data recording and genetic evaluation systems are the community-based breeding programmes (CBBP) for sheep and goats in Ethiopia run by ICARDA and partners where data are collected using AniCapture tool and stored at AniCloud database (https://anicloud.com), and the CBBP for goats in Tanzania, Uganda and Malawi. The successful data recording by ADGG and the CBBP programmes were due to innovative use of mobile technologies and ICT tools as reviewed by Mrode et al. (2020). The adoption of the ADGG approach to fit the specific local country needs, realities and environments in sustainable ways is needed. Therefore, understanding and embracing the partnerships between farmers/farmer organizations, sub-national, national, key private sector actors, local and national governments as well as the development partners are critical to the success of the on-going data recording systems in Africa and would be expanded to other programs.

The challenge of inadequate and lack of human capacity to undertake genetic evaluation is real (Rege et al., 2022). The adoption of mass selection methods by the majority of African animal breeders is partly due to the lack of animal identification, human capacity to handle pedigree and genomic data as well as the limited financial resourcing of livestock improvement programmes. In the present study, in regions where human capacity is lower there was a high dominance of phenotypic performance-based selection compared to pedigree-based and genomic evaluations. Furthermore, sub-regions with higher available livestock data (Eastern, Western, and Southern) lack human capacity to carry out livestock genetic evaluation. Past efforts to tackle the human capacity challenge has included the development of animal genetic training resources and “training the trainers” programme where more than 100 scientists from 25 countries in sub-Saharan Africa and 15 countries in South and South East Asia were trained through workshops and refresher courses (Ojango et al., 2009). The recently established African Animal Breeding Network (AABNet, http://animalbreeding-africa.org/) could also build on previous efforts to address the human capacity issue through annual and purposely designed short term training courses in animal breeding, quantitative genetics, genomics and bioinformatics. The tailor-made short courses will emphasize theoretical concepts, problem-solving and hands-on training. It is envisaged that, in addition to strengthening of the capacity of scientists AABNet will work with strategically selected individual countries to support the development of long-term genetic improvement programmes, including performance recording, pedigree information, genotyping to support genetic analysis, genomic prediction, *inter alia*. The case farms identified in target countries as part of the initiative will be supported by the trained scientists who will help them to select and disseminate genetically superior animals during implementation phase of breeding programmes. Systems for animal performance recording that provide feedbacks to farmers need to be developed with supportive policies to enable their large-scale adoption (Opoola et al., 2019). In southern and eastern African regions, it has been reported that institutional set up to support animal breeding programmes is fragmented and that livestock recording for the purpose of research and development breeding practices is lacking (Zonabend et al., 2013). Similar issues have been observed in the other African sub-regions in the present study. There is therefore a need for collaboration between countries and regions to tackle the common issues hindering livestock genetic improvement in the continent. This will require that African governments commit to setting aside a certain percentage of their livestock budgets to purchase state-of-the-art equipment and upgrade the existing ones with strong institutional support. At the same time there is need to strengthen livestock policies and ensure that they are adequately implemented.

### 4.2 Potential for across country genetic evaluation

Most of the respondents believe that across-country genetic evaluation in Africa will have an important mileage in livestock sector. Across-country evaluation will build genetic evaluation capacities of countries where such capacities are lacking. This will also partly solve the resource challenge as by pooling resources, the countries with inadequate resources can be supported by those who have, including international institutions (e.g., ICAR, AABNet, and ADGG). In addition to solving the inadequacy of human capacity, across country evaluation will enable more rigour and higher reliabilities of the genetic predictions. For example, the current study showed that Kenya benefitted more from selecting sires across different countries than using only its own national sires for genetic evaluation of MY305 and AFC while South Africa benefitted for AFC and CI1. The benefits from across country genetic evaluations in cattle have been well demonstrated in developed countries (Banos and Smith, 1991; Hammami et al., 2009; Nilforooshan, 2011). In a recent study in Sub-Saharan Africa, Opoola et al. (2020) utilized the across country method to examine MY305, AFC and CI1 in Holstein-Friesian and Jersey cattle breeds in Zimbabwe, South Africa and Kenya. Results showed that the genetic variance and heritability were not always estimable within-country but were significantly different from zero in the across country evaluation, and there was greater predicted genetic gains in all traits from the across-country genetic evaluation due to greater accuracy of selection compared to within-country. However, Opoola et al. (2020) used single trait animal model in contrast to the multi-trait analysis implemented in the present study. The multi-trait analyses take into consideration the genetic correlation between the studied traits. Furthermore, all previous attempts of across county genetic evaluation assumed a genetic correlation of unity and did not take into consideration the genotype-by-environment effect as the classical method implemented by Interbull. The greater predicted genetic gains in across country evaluation is due to the existence of genetic links across countries. For the computation of genetic gains only the sire pathways have been considered. Therefore, the rate of genetic gain reported in the present study represents approximately about 66% of possible genetic progress (Schmidt and Vleck, 1974). This implies that the benefit from across country genetic evaluation would even be higher if cows were also selected. Across country genetic evaluation requires strong expertise and collaborations (Opoola et al., 2020). Unfortunately, South Africa is the only African country participating in the Interbull of international Committee for Animal Recording (ICAR). Efforts need to be made to get other African countries to join the Interbull international genetic evaluation.

As alluded to above, a multi-country breeding programme based on joint genetic evaluation would be possible when there are genetic links across countries, and would provide a platform for accelerated genetic gains through selection and germplasm exchange between sub-Saharan African countries (Opoola et al., 2020). However, the lack of herd connectedness and pedigree data recording in African traditional production systems may limit the application of the classical multiple across country genetic evaluation in African indigenous livestock. This is where the use of genomic methodologies would be very useful. For example, a genomic matrix can be used to assess relationships allowing estimation of genomic breeding values to enable selection of superior parents to drive improvement. Genomic selection also offers the advantage of selecting young animals and hence reducing generation interval compared to the traditional approach. Therefore, across-country genomic evaluation could be the way for across country genetic evaluation in African indigenous livestock breeds. A recent study, has tested the feasibility of multiple country and breed genomic prediction of tick resistance in seven beef cattle breeds including the South African indigenous Nguni cattle breed (Cardoso et al., 2021). The results showed that genomic multi-traits approach improved predictive ability for resistance to ticks and could be used to improve tick resistance of the studied populations (Cardoso et al., 2021). Moreover, the ADGG programme is generating data across Ethiopia, Tanzania, Kenya, Uganda, and Rwanda using genomic evaluation methods and could serve as a testbed for multiple across country genomic evaluation in Africa. A recent study examining ADGG data between Ethiopia and Tanzania indicated a very low genetic correlation of about 0.13 for milk yield between the two countries and highlighted the need to deliberately exchange top ranking bulls between the countries (Mrode et al., 2022). Furthermore, the present study revealed the existence of livestock performance (milk traits, growth, reproduction, health, etc.), pedigree, genetic information data on various cattle breeds across African countries and sub-regions as well as the custodians of these data. As a next step, AABNet could put in place a Memorandum of Understanding between data custodians across countries. The governments and custodians of the available data in each country should be sensitized about the benefits of across country genetic evaluation and about sharing resources and capacity to carry out across-country genetic evaluation for common traits as done in western countries. The diversity displayed by African indigenous livestock breeds as well as the existence of capacity in the African diaspora already involved in the ICAR international genetic evaluation, and the establishment of AABNet, are important opportunities to be exploited to rapidly promote genetic improvement between African countries. Although the difference in breeding goals between countries has been listed as a challenge by respondents, scenarios can be assumed and analyses that speak to the similarities can be undertaken. A sustainable animal breeding programme in Africa would require a strong national and regional collaboration and collective actions by all the stakeholders (farmers, breed societies, research institutes, universities, governments, private businesses, and NGOs) working together to achieve a common goal as illustrated by Ibeagha-Awemu et al. (2019).

### 4.3 Future approaches for livestock phenomics and genetic evaluation in Africa

Application of ICT and mobile devices to record performance data on-farm in dairy cattle and small ruminants is already happening in Africa (Mrode et al., 2020). In South Africa successful attempts have been made in the use of mobile platforms and low-cost censors for precision phenotyping in both beef and dairy cattle (Visser et al., 2020). The current ICT and mobile technologies used for livestock data recording are still relying on internet which is a big challenge in Africa. Going forward, livestock phenomics and genetic evaluation in the continent will need to include digital tools and ICT that do not rely on internet (Mrode et al., 2020). Availability of requisite human capacity, infrastructure, appropriate animal breeding policies, and adequate financial resources will be required to underpin functional and sustainable genetic evaluation systems. These modest successes to date, have been made by countries working independently, mostly in donor-funded, time-bound projects. There is a need to build on these efforts to establish a continental initiative that leverages on potential complementarities and synergies. Moreover, most the traits currently being recorded are easy to measure. These include milk yield, live weights based on heart girth, body condition score, growth traits, age at calving and calving interval. The more difficult traits to measure such as residual feed efficiency, feed conversion ratio and disease resistance are yet to be recorded using the mobile technologies. There will be need to develop harmonized standards for livestock identification and precision phenotyping using ICAR as a reference. Given that there are already many Apps that are being used to collect data from farmers’ herds, there is a need for collaboration around data collection and sharing using agreed protocols and standards, and governed by formalized agreements, first within countries, and deploying more robust management tools (e.g., block chains) and automation of database links via APIs (Application Programming Interface).

There is also scope for inclusion of georeferencing of herds to enable connection of collected performance data with related global meta-weather data to estimate the effects of climatic conditions on productivity and hence integrate climate resilience in livestock breeding objectives. In addition, a combination of mobile telephony and collection of e GPS coordinates of farms can provide for a powerful spatial modelling to identify superior animals in smallholder farms (Selle et al., 2020), thus overcoming some of the current challenges associated with low connectedness among farms which are also widely scattered and difficult to reach. AABNet could play an important role in advancing the livestock phenomics and genetic evaluation agenda in the continent by facilitating the development of harmonized livestock identification and data recording systems for the willing African countries and supporting them to carry out their national routine genetic evaluation.

Availability of appropriate technical capacity is crucial for functioning genetic improvement programmes. Many African University curricular have not been responsive to the rapid developments in technologies and the opportunities these provide for animal genetic evaluations and overall design and execution of breeding programmes. As alluded to above, the mission of AABNet places it in good place to facilitate the retooling of African animal geneticists and breeders as well as private sector players, including farmers, to equip them with what they need to drive genetic improvement programmes using state-of-the art technologies and tools. One tool approach in this regard could be through organisation of massive open online courses (MOOCs) and annual summer classes. The key topics of the training sessions will focus on definition of breeding goals adapted to African livestock production system systems, hands-on training on inclusion of performance, pedigree, and genomic information in the estimation of genetic merit of animals, breeding programme design, optimisation and simulation. This will help to develop breeding programmes that are sustainable and adapted to the various livestock production systems in the continent. AABNet has recently organised a 3-week training (14 February—11 March 2022) to support 48 participants from 30 African countries. The training mode used included classroom lectures, and hands-on lab sessions covering various topics. A big emphasis on definition of standard and harmonisation animal identification and data recording systems will be needed in future trainings. A lesson learnt from the previous trainings organised in Africa is non-availability of real data from the attendees as well as the short duration of the trainings limiting the acquisition of hands-on skills. To address this challenge, AABNet could invite the participants from the institutions of the custodians of available livestock data (as reported in this study). The custodians of data could nominate participants from their institutions who would bring specific data sets with them to analyse as part of the training. This approach can potentially deliver huge benefits by contributing to the transfer of the acquired skills to the home organisations. After the national genetic evaluation, the across country genetic evaluation could be carried out under the coordination and technical support by the AABNet members—both in the continent and Diaspora—and the results shared with participating countries through processes which also include capacity development to enhance effective use of the results. For across country evaluation, emphasis should initially be on dairy cattle due to the evidence of use of international sires for artificial insemination in different African countries. As also mentioned by Mulder et al. (2017), the most critical point in establishing cross-country collaboration in the African context is to create an environment of fairness and equity in data and benefit sharing. This should be an important consideration from the start of discussions among partners from the different countries, and formal agreements should have clear statements of ownership and benefits.

## 5 Conclusion

This study has highlighted the current status of livestock phenomics and genetic evaluation approaches in Africa, with a focus on the main challenges at the national, sub-regional and continental levels, and possible solutions to these challenges. The challenges identified are related to animal identification and data recording systems, availability of digital tools and ICT not relying on internet, human capacity, infrastructure, funding and animal breeding policies. It is evident that the lack of a robust animal identification and data recording systems as well as human capacity has greatly influenced the choice of selection method, and explains the predominance of mass selection as the method currently being applied by most countries. A case example done as part of this study of joint genetic evaluation of Holstein-Friesian cattle data from Kenya and South Africa resulted in higher accuracy of prediction and genetic gains, demonstrating the benefits of such an approach. Results showed that Kenya benefited from the joint evaluation on the 305-days milk yield and the age at first calving, while South Africa got benefits on the age at first calving and the first calving interval. In addition to demonstrating these benefits, the findings of this study have identified issues around harmonized protocols for animal identification, livestock data recording and genetic evaluations (both national and across-country) as well as capacity building and training programmes for animal breeders and livestock farmers in Africa. Other needed enablers identified include policies, appropriate infrastructure and funding. It is concluded that the development of a joint genetic evaluation across African countries could revolutionize livestock genetic improvement in the continent.

## Data Availability

The raw data supporting the conclusion of this article will be made available by the authors, without undue reservation.
