# Supplementary material for: Livestock phenomics and genetic evaluation approaches in Africa: current state and future perspectives
Source: Front Genet. 2023 Jun 8;14:1115973. doi: 10.3389/fgene.2023.1115973 (PMC10285055; doi:10.3389/fgene.2023.1115973)
Supplement: Supplementary file 1 [file Table1.docx]

**Supplementary Table S1: Distribution of survey responses by country, gender, occupation and livestock species**

| Country | Region | N° of Respondents | Gender | | Occupation | Livestock species |
| --- | --- | --- | --- | --- | --- | --- |
|  |  |  | Female | Male |  |  |
| Algeria | Northen | 2 | 0 | 2 | One Government researcher and one university lecturer | Sheep, Goat, Chicken, Dog, and Camel |
| Angola | Southern | 1 | 0 | 1 | University lecturer | Goat |
| Benin | Western | 5 | 0 | 5 | Goverment (One extension worker, One lecturer and Three university researchers) | Cattle (Dairy), Cattle (Beef), Sheep, Goat, Pig and Chicken |
| Burkina Faso | Western | 3 | 0 | 3 | Two Government researchers and One Sub-regional Research Organization researcher | Cattle (Dairy),Sheep, Goat, Pig, Chicken and Guinea fowl |
| Cabo Verde | Western | 1 | 1 | 0 | Government extension worker | Cattle (Dairy), Sheep, Goat, Pig and Chicken |
| Cameroon | Central | 4 | 0 | 4 | One Government extension worker, Two lecturers and One student | Cattle (Dairy), Cattle (Beef), Sheep, Goat, Pig, Chicken, Guinea fowl, Cavy and Duck |
| Chad | Central | 1 | 0 | 1 | Breeders Associations/Farmers cooperatives | Guinea fowl |
| Comores | Eastern | 1 | 0 | 1 | Non-governmental organisation researcher | Cattle (Dairy), Cattle (Beef), Sheep, Goat and Chicken |
| Congo | Central | 2 | 0 | 2 | One Government researcher and One University researcher | Chicken and Duck |
| Côte d'Ivoire | Western | 5 | 0 | 5 | Government (Three lecturers, One Government Researcher and One University researcher | Cattle (Dairy), Cattle (Beef)   Chicken, Cavy, Rabbit and Grasscutter |
| Djibouti | Eastern | 1 | 0 | 1 | Government extension worker | Goat |
| DR Congo | Central | 2 | 0 | 2 | Two university lecturers | Cattle (Dairy), Cattle (Beef), Goat, Chicken and Cavy |
| Egypt | Northen | 4 | 1 | 3 | Two Government researchers and Two professors | Cattle (Dairy), Cattle (Beef), Sheep, Goat, Chicken, Rabbit, Camel and Water buffalo |
| Ethiopia | Eastern | 3 | 0 | 3 | One International Research Organisation Researcher, One government researcher and One University researcher | Cattle (Beef), Sheep and Goat |
| Gabon | Central | 1 | 0 | 1 | University lecturer | Sheep, Pig, and Chicken |
| Ghana | Western | 1 | 0 | 1 | University lecturer | Cattle (Dairy), Cattle (Beef), Sheep, Goat, Pig and Chicken |
| Kenya | Eastern | 2 | 0 | 2 | One Government researcher and one university lecturer | Cattle (Dairy), Cattle (Beef), Sheep and Chicken |
| Liberia | Western | 1 | 0 | 1 | Non-governmental organisation researcher | Cattle (Beef), Sheep, Goat, Pig and Chicken |
| Malawi | Southern | 2 | 0 | 2 | University lecturer | Cattle (Dairy), Cattle (Beef), Goat, Pig and Chicken |
| Mali | Western | 1 | 0 | 1 | Government researcher | Cattle (Dairy) |
| Mauritania | Northen | 1 | 0 | 1 | University lecturer | Cattle (Dairy), Cattle (Beef), Sheep, Goat, Chicken, Camel, Horse and Donkey |
| Morocco | Northen | 2 | 0 | 2 | One Government development worker and One University lecturer | Cattle (Dairy), Cattle (Beef), Sheep, Goat and Horse |
| Mozambique | Southern | 1 | 1 | 0 | Government researcher | Cattle (Beef), Pig and Chicken |
| Niger | Western | 2 | 0 | 2 | One Government development worker and One student | Cattle (Dairy), Cattle (Beef), Sheep, Goat, Pig, Chicken and Guinea fowl |
| Nigeria | Western | 11 | 3 | 8 | Three Government Researcher, Six university lecturer, Two university researcher | Cattle (Dairy), Cattle (Beef), Sheep, Goat, Pig, Chicken, Guinea fowl, Turkey and Rabbit |
| Rwanda | Eastern | 2 | 0 | 2 | One university lecturer and One Government researcher | Cattle (Dairy), Cattle (Beef), Pig and Chicken |
| Senegal | Western | 3 | 0 | 3 | One Government researcher and Two University lecturer | Cattle (Dairy), Cattle (Beef) and Sheep |
| Seychelles | Eastern | 1 | 0 | 1 | Government researcher | Pig |
| Sierra Leone | Western | 1 | 0 | 1 | University lecturer | Cattle (Beef), Sheep, Goat, Pig and Chicken |
| South Africa | Southern | 5 | 2 | 3 | Government Research Manager & Policy maker, Two Government researchers and Two university lecturers | Cattle (Dairy), Cattle (Beef), Sheep, Goat, Pig and Chicken |
| Sudan | Eastern | 1 | 1 | 0 | University researcher | Cattle (Dairy) and Goat |
| Tanzania | Eastern | 4 | 0 | 4 | Government researcher | Cattle (Dairy), Cattle (Beef), Sheep, Goat, Pig and Chicken |
| The Gambia | Western | 2 | 0 | 2 | Government (One researcher and One Development worker) | Cattle (Dairy), Cattle (Beef), Sheep and Goat |
| Togo | Western | 2 | 0 | 2 | One University lecturer and One Government researcher | Cattle (Beef), Sheep, goat, Chicken, Guinea fowl |
| Tunisia | Northen | 2 | 1 | 1 | One Government researcher and One University lecturer | Cattle (Dairy), Sheep, Goat, Horse and Camel |
| Uganda | Eastern | 6 | 1 | 5 | One University lecturer, One Government (Extension) officer and four Government researchers | Cattle (Dairy), Cattle (Beef), Goat, Pig, Chicken and Guinea fowl |
| Zambia | Southern | 1 | 0 | 1 | University lecturer | Cattle (Dairy), Cattle (Beef), Goat, Chicken and Rabbit |
| Zimbabwe | Southern | 2 | 0 | 2 | One Freelance consultant and One Government researcher | Cattle (Dairy), Cattle (Beef), Sheep, Goat, Chicken and Rabbit |
| Total |  | 92 | 11 | 81 |  | |
| % |  | 100 | 12 | 88 |  |  |
