# Supplementary material for: Livestock phenomics and genetic evaluation approaches in Africa: current state and future perspectives
Source: Front Genet. 2023 Jun 8;14:1115973. doi: 10.3389/fgene.2023.1115973 (PMC10285055; doi:10.3389/fgene.2023.1115973)
Supplement: Supplementary file 2 [file DataSheet1.PDF]

African Animal Genetic Improvement and Evaluation Scoping Study

The research aims at examining current genetic improvement techniques and genetic evaluations being carried out in African countries, the available livestock data and potential for multi-countries collaborations for livestock genetic improvement in Africa. We would be very grateful if you would complete this questionnaire (In English or French). This will take you about 15 minutes.

* Required

1. Email address *

____

2. Are you a male or female? *

Mark only one oval.

- ( ) Male
- ( ) Female

3. In which country are you based? *

____

4. What kind of organization do you work with? *

Mark only one oval.

- Government (Extension)
- Government (Research)
- University
- Non-governmental organisation (NGO)
- Private company
- Breeders Associations/Farmers cooperatives
- Other: ____________________

5. How will you classify your position? *

Mark only one oval.

- Lecturer
- Student
- Researcher
- Development worker
- Extension worker
- Other: ____________________

6. Which livestock specie (s) do you primarily work on? *

Check all that apply.

- [ ] Cattle (Dairy)
- [ ] Cattle (Beef)
- [ ] Sheep
- [ ] Goat
- [ ] Pig
- [ ] Chicken
- [ ] Guinea fowl

Other: [ ] ____________________

7. What are the main breeds and breed combinations of the species you primarily work on? Please list them by specie *

8. Is there any livestock genetic improvement program/project ongoing with both performance and pedigree/genotypic data recorded in your country? *

Mark only one oval.

- Yes *Skip to question 9*
- No *Skip to question 18*

Livestock data available

9. If yes from which species these data are recorded? *

Check all that apply.

- [ ] Cattle (Dairy)
- [ ] Cattle (Beef)
- [ ] Sheep
- [ ] Goat
- [ ] Pig
- [ ] Chicken
- [ ] Guinea fowl

Other: [ ] ____________

10. What type of data is mainly collected and available? *

Check all that apply.

- [ ] Milk traits data
- [ ] Carcass and meat traits
- [ ] Growth traits
- [ ] Reproduction data
- [ ] Herd health data
- [ ] Pedigrees
- [ ] Genomic data (high density platform)
- [ ] Genomic data (low density platform)
- [ ] Economic data

Other: [ ] ________________

11. For how many generations and/or years these data are recorded? *

____

12. What are the selection methods being used within the programme/project? *

Check all that apply.

- [ ] Based on genetic evaluation
- [ ] Based on genomic evaluation
- [ ] Based on phenotypic performance data

Other: [ ] ________________

13. Please, describe the selection methods being used within the Programme/project *

____
____
____
____
____

14. Who are the keepers (custodians) of the available data? *

Check all that apply.

- [ ] Databases,
- [ ] Government
- [ ] NGOs
- [ ] Research institutes

Other: [ ] ______________________

15. Do you think it's a good idea to share the data and will it be possible? *

Mark only one oval.

- ( ) Yes
- ( ) No

16. Would the custodians of such data be willing to be supported to use the same data to undertake better genetic evaluation and explore possibility of genomic evaluations and joint genetic evaluation across African regions? *

Mark only one oval.

- ( ) Yes
- ( ) No
- ( ) Maybe

17. Please give address of key contact person (s) of the custodians: name, institution, email and phone (optional) *

____

____

____

____

____

Untitled Section

18. Is there a systematic national animal identification system in your country? *

Mark only one oval.

- ( ) Yes *Skip to question 19*
- ( ) No *Skip to question 20*

Animal identification systems

19. If yes please describe the national animal identification system *

____
____
____
____
____

Untitled Section

20. What are the different types of animal identification used at the herd level? *

Check all that apply.

- [ ] Ear tagging
- [ ] Ear Tattooing
- [ ] Number tagging
- [ ] Branding
- [ ] Ear Notching

Other: [ ] ____________________

21. Is there a national livestock selection/ ranking system? *

Mark only one oval.

- ( ) Yes *Skip to question 22*
- ( ) No *Skip to question 25*

National animal selection/ranking system

22. If yes, please indicate the species *

Check all that apply.

- [ ] Cattle (Dairy)
- [ ] Cattle (Beef)
- [ ] Sheep
- [ ] Goat
- [ ] Pig
- [ ] Chicken
- [ ] Guinea fowl

Other: [ ] ____________________

23. What are the methods used for the national livestock selection/ ranking? *

Check all that apply.

- [ ] Based on genetic evaluation
- [ ] Based on genomic evaluation
- [ ] Based on phenotypic performance data
- [ ] Other selection criteria (Please describe in next question)

24. Describe the procedure of the evaluation method(s) you chose in the previous question *

____
____
____
____
____

Untitled Section

25. Are there any deliberate human capacity building initiatives in animal science/animal breeding in your country? *

Mark only one oval.

- Yes
- No

26. In your opinion, what are the main challenges that are affecting livestock genetic evaluation in the country where you are based? List them in order of priority starting with the highest. *

27. In your opinion, what are the measures that can be undertaken to mitigate those challenges? *

28. Across-country genetic evaluation is a joint genetic evaluation between countries. This scheme has been successful in developed countries. Would you think that such evaluation scheme will have some mileage in improving livestock production in Africa? *

Mark only one oval.

- Yes
- No

Untitled Section

29. Please give reason (s) ? *

30. In your opinion, what may be the challenges of the implementation of across-country genetic evaluation scheme in African livestock production systems? List them in order of priority starting with the highest. *

31. Are there any other comments/ suggestions that you think could help in livestock genetic improvement in the country where you are based? Any lessons learnt from previous genetic improvement programs?

This content is neither created nor endorsed by Google.

Google Forms
